# Reading vertically and horizontally mirrored text: An eye movement investigation

**DOI:** 10.1177/17470218221085943

**Published:** 2022-04-20

**Authors:** Katharina Pittrich, Sascha Schroeder

**Affiliations:** Department of Educational Psychology, University of Göttingen, Göttingen, Germany

**Keywords:** Mirrored text, eye movements, reading

## Abstract

This study examined the cognitive processes involved in reading vertically and horizontally mirrored text. We tracked participants’ eye movements while they were reading the Potsdam Sentence Corpus which consists of 144 sentences with target words that are manipulated for length and frequency. Sentences were presented in three different conditions: In the normal condition, text was presented with upright letters, in the vertical condition, each letter was flipped around its vertical (left-right) axis while in the horizontal condition, letters were flipped around their horizontal (up-down) axis. Results show that reading was slowed down in both mirror conditions and that horizontal mirroring was particularly disruptive. In both conditions, we found larger effects of word length than in the normal condition indicating that participants read the sentences more serially and effortfully. Similarly, frequency effects were larger in both mirror conditions in later reading measures (gaze duration, go-past time, and total reading time) and particularly pronounced in the horizontal condition. This indicates that reading mirrored script involves a late checking mechanism that is particularly important for reading a horizontally mirrored script. Together, our findings demonstrate that mirroring affects both early visual identification and later linguistic processes.

Reading is a complex cognitive activity that involves both visual and language-related processes. During this process, letters have to be discriminated and mapped onto words. However, letters are rather unique visual stimuli that differ in many ways from other perceptual objects. On one hand, the letter identification system has to be flexible so that visual objects which vary in size, position, or shape are recognised as instances of the same entity (e.g., “a,” “A,” “*a*,” “A,” “a”). On the contrary, the system has to be sensitive enough in order to register even small discrepancies between letters, e.g., the difference between the letter “c” and “e” (Dehaene et al., 2005; Grainger et al., 2008). The question of how humans accomplish this remarkable task is still unresolved. To answer this question, investigating how people read mirrored script, i.e., letters that are presented upside down or flipped around their vertical axis, is particularly informative. In the natural world, most objects are usually perceived as the same irrespective of the observer’s viewpoint (Corballis & Beale, 1976). Letters, as human-made artefacts, are different though. Although most ancient alphabets had flexible reading directions, the lower case letters of the Latin alphabet were designed to be read from left-to-right and from the top to the bottom of a page (Wallace, 2012). As a consequence, some letters, most notably “b,” “d,” “p,” and “q,” receive radical different interpretations although they have the same shape and differ mainly in their orientation. Dehaene et al. (2005) suggested that efficient reading requires the suppression of mirror-invariance that is present in other perceptual domains.

Although mirror-invariance and mirror-image confusions can be a special impediment in reading the alphabetic script, studies investigating readers’ eye-movement behaviour during the reading of mirrored text are still scarce (but see Chandra et al., 2020; Kowler & Anton, 1987). In particular, the cognitive mechanisms underlying reading text with mirrored letters are still unknown, and it remains unclear which processes are affected by mirroring. To address this question, we conducted an eye-tracking experiment in which adults read the Potsdam Sentence Corpus (Kliegl et al., 2004), a well-researched eye-tracking corpus in which target words are manipulated according to their length and frequency. Participants read the sentences in three different mirror conditions, a normal condition and two mirror conditions in which individual letters were either mirrored around their vertical (left-right) or horizontal (up-down) axis. We were especially interested in the effect of mirroring on participants’ word length and frequency effects. In particular, we wanted to see whether the effects of word length and frequency would interact with mirror condition to determine which stage of the word recognition process is affected by mirroring (Staub, 2020).

## Introduction

The time that a reader spends processing a sentence or word reflects the ease with which text is processed. It is well established that both linguistic and visual variables affect how readers navigate their eye movements when reading connected text (Liversedge & Findlay, 2000; Rayner, 1998, 2009; Rayner & Duffy, 1986). The two most prominent linguistic variables are word frequency and predictability. Words that occur more frequently in a language are processed faster and skipped more frequently (Inhoff & Rayner, 1986; Kliegl et al., 2006; Rayner & Duffy, 1986). Similarly, reading times are longer on a word that is unpredictable given its preceding context (Altarriba et al., 1996; Balota et al., 1985; Rayner et al., 2004). Both effects are benchmark findings in the eye-movement literature and demonstrate that reader’s eye movements are tightly connected to the cognitive processes involved in language comprehension. As a consequence, they have both been incorporated in computational models of eye movements in reading such as E-Z Reader (Reichle et al., 1998, 1999) or SWIFT (Engbert et al., 2002).

Next to these language-related variables, a number of studies have investigated variables that are assumed to affect the visual processes involved in reading. These include contrast reduction (Reingold & Rayner, 2006; Sheridan & Reingold, 2013; Staub, 2020; White & Staub, 2012), font difficulty (Rayner et al., 2006; Staub, 2020), cAsE aLtErNaTiOn (Drieghe, 2008; Juhasz et al., 2006; Reingold & Rayner, 2006), or letter rotation (Blythe et al., 2019). These studies have shown that such visual manipulations make reading more difficult although to varying degrees. For example, while the effects of contrast reduction are relatively subtle (increasing sentence reading times 5%–20%; e.g., Warrington et al., 2018), letter rotations can have a massive impact on the reading process, especially if the rotation angle exceeds 60º and the rotation direction alternates between letters (which increases sentence reading times by ca. 300%; e.g., Blythe et al., 2019).

Surprisingly, although reading mirrored script has attracted a substantial amount of attention in cognitive science (Chandra et al., 2020; Kowler & Anton, 1987; Rabe et al., 2021) and neuroscience (Kassubek et al., 2001; Poldrack et al., 1998; Poldrack & Gabrieli, 2001; Ryan & Schnyer, 2007), studies investigating readers’ eye movements while reading mirrored script are relatively scarce and have mainly focused on saccade targeting. For example, Kowler and Anton (1987) investigated the eye movements of two participants who read texts in which individual letters, words, or whole texts were either vertically or horizontally mirrored. They found that mirroring individual letters increased reading times dramatically from ca. 60 ms/letter during normal reading to ca. 240 ms/letter in the vertical mirror condition and ca. 460 ms/letter in the horizontal mirror condition. As the authors were mainly interested in the effects of mirroring on saccade programming, word reading times were not analysed. However, the authors reported that mean saccade length was much smaller during the reading of mirrored text, indicating that words were decoded serially and less fluently.

This pattern is consistent with the results of several behavioural studies that have been conducted in the 1960s and 1970s. For example, in a seminal study, Kolers (1968) investigated participants’ reading speed as they read the text in which either single letters or the whole text was vertically or horizontally mirrored. In addition, he also manipulated the reading direction (left-to-right, right-to-left). Findings showed that reading performance was mainly driven by an interaction of mirror condition and reading direction. Just focusing on the results in the left-right reading direction and mirroring individual letters, results showed that reading speed decreased from 220 words/min during normal reading to ca. 100 words/min in the vertical mirror condition and ca. 50 words/min in the horizontal reading condition.

In a recent eye-movement study, Chandra et al. (2020) investigated various eye-movement measures during the reading of vertically mirrored text. They particularly focused on the impact on oculomotor processes and the eyes’ landing positions. Participants read texts in a normal reading condition as well as when the individual letters and/or the entire words were written from right-to-left. For the letter condition, they reported that mirroring increased mean fixation duration from ca. 250 ms to ca. 300 ms. They also found that vertical mirroring decreased the proportion of skippings (from 30% to 13%) and regressive saccades (from 12% to 7%) and increased the number of refixations (from 23% to 30%). Similar to Kowler and Anton (1987), they found that forward saccade length was shorter in mirrored reading and the distribution of initial landing positions was shifted to the beginning of the word. Unfortunately, Chandra et al. (2020) did not include a horizontal mirror condition in their experiment. In addition, they did not investigate word length and frequency effects.

In sum, previous studies suggest that reading mirrored text substantially slows down reading and that vertical mirroring is less disruptive than horizontal mirroring. The main reason for this slow down is that parallel word identification breaks down and readers employ a serial processing strategy that impedes the reading process.

If mirrored text is read more serially, one would expect that mirroring interacts with word length. If the letters in a word are processed individually from left to right, reading times should increase linearly with the number of letters in a word. Word length effects are a marker effect for the influence of serial, attention-demanding letter-by-letter processing (e.g., Perry & Ziegler, 2002). This view is supported by the findings from single-word recognition studies that have investigated the effect of mirroring using the naming or the lexical decision task. For example, Björnström et al. (2014) investigated word-length effects when participants read aloud words of various lengths that were presented normally or, among other conditions, horizontally mirrored. They found that word length effects increased substantially from ca. 13 ms/letter to ca. 550 ms/letter. A similar interaction between word length and presentation mode has also been reported for rotated text by Navon (1978).

Indeed, adults reading mirrored text show a similar reading pattern as beginning or less-skilled readers. This is the reason why mirror reading has been used in many learning studies (Kassubek et al., 2001; Kolers, 1968, 1976; Kolers & Perkins, 1975; Poldrack et al., 1998). In these studies, adults who are highly proficient in reading normal text learn to read mirrored text. As elaborated above, this is initially very demanding, but the reading process quickly becomes more automatic with increasing training. For example, Kolers and Perkins (1975) showed that readers were able to read horizontally mirrored text with near-normal speed after having read ca. 100 pages of mirrored text. Similarly, functional magnetic resonance imaging (fMRI) studies show structural changes in reading-related brain regions even after one training session (Poldrack et al., 1998). The learning curve follows a standard power function (Newell & Rosenbloom, 1993) indicating that letter identification becomes increasingly more parallel during training (Logan, 1988).

While it is clear that reading mirrored text is more resource-demanding, it is not entirely clear why this is the case. Possible mechanisms are that mirrored letters consist of visual features that are less frequent or familiar to the reader or that these features are combined differently in mirrored letters. As a consequence, new feature-letter mappings have to be established. In terms of the interactive-activation model of visual word recognition (McClelland, 1979), mirroring effects are likely to occur on the feature-level or involve feature-to-letter mappings. This view is consistent with the Local Combination Detector model that has been proposed by Dehaene et al. (2005). The model assumes a hierarchy of neural populations in the occipito-temporal visual pathway in which basic visual elements are successively combined into higher perceptual units and increasingly larger fragments of a word. Within this model, mirroring most likely affects the connections between local letter features (which are processed in V2), case-specific letter shapes (which are processed in V4), and a bank of abstract letter detectors (located at V8). In line with this model, fMRI studies show strong associations between mirror reading and activity in the so-called visual word form area (VWFA) within the left occipito-temporal cortex (Cohen et al., 2008; Poldrack et al., 1998).

Another question that remains unresolved is why horizontal mirroring slows down the reading process more than vertical mirroring. One possible explanation is that vertical and horizontal mirror reading rely upon different cognitive mechanisms. This view is supported by an fMRI study of Zhao et al. (2010) who showed that reading horizontally mirrored compared with upright Chinese characters shifted the fMRI response from the VWFA towards regions involved in generic objects processing. Similarly, the processing of vertically mirrored letters (i.e., <b> vs. <d> ) is likely to differ from the effects of other very similar visual transformations such as plane rotations (i.e., <b> vs. <q> ) because vertical mirror-image discrimination and the discrimination of rotated images have been found to rely upon selective cognitive processes and anatomical networks (Logothetis et al., 1995; Martinaud et al., 2016).

Within the letters of the Latin alphabet, there are several distinctive features that have been identified as being crucial for letter identification. These features include terminations, straight lines, curved lines, oblique lines, and intersections (Briggs & Hocevar, 1975; Fiset et al., 2008; Gibson, 1969). Curved lines are found in many letters and they are mostly oriented vertically (i.e., <a> , <b> , <c> , <d> , <e> , <g> , <p> , <q> ) whereas downward- or upward-oriented curved lines are rather rare (i.e., <u> , <n> , <m> ). Vertical mirroring thus likely disrupts the recognition of particularly those letters which comprise vertically oriented curved lines. By contrast, horizontal mirroring likely disrupts the recognition of letters that comprise ascending or descending straight lines for which the up-down orientation is a diagnostic feature (i.e., <b> , <d> , <f> , <g> , <h> , <j> , <p> , <q> , <t> ). Given that there are about as many letters with vertically oriented curved lines as there are letters with ascending and descending straight lines, some degree of left-right mirror-invariance may account for why vertically mirrored text is easier to read than horizontally mirrored text (e.g., Corballis & Beale, 1976; Dehaene et al., 2005; Rollenhagen & Olson, 2000). However, it is also plausible to believe that in the horizontal mirror-condition, interference effects are stronger because there are more letters which are prone to be mirror-confused with a different normal letter when mirrored horizontally (i.e., <b> , <d> , <p> , <q> , <n> , <u> , <w> ) compared to when mirrored vertically (i.e., <b> , <d> , <p> , <q> ) (we come back to this point in the discussion section).

While it is plausible that mirroring affects early visual letter processing, it is less clear whether it also affects later language-mediated processes. To investigate this question, we also examined the effects of mirroring on frequency effects which are a marker effect of lexical processing (Rayner, 2009). According to Sternberg’s additive factors logic (Sternberg, 1969), two variables that have additive effects affect different stages of processing, whereas two variables that have interactive effects may affect at least one common stage of processing. Applying this logic to the present study, an interaction between mirroring and word frequency would support the assumption that mirroring affects lexical processing. However, if the two variables were to produce additive effects, this finding would support the interpretation that mirroring effects are confined to visual letter-processing.

The question of whether mirroring and word frequency have additive or interactive effects has not yet been investigated empirically. However, there are many studies that have examined the interaction between word frequency and other visual variables (Jainta et al., 2017; Liu et al., 2016; Rayner et al., 2006; Reingold et al., 2010; Sheridan & Reingold, 2013; Staub, 2020; Warrington et al., 2018). The empirical picture is mixed, but most studies that manipulated stimulus quality via contrast reduction or blurring found additive effects (Staub, 2020). By contrast, manipulations that target letter-level processing such as font difficulty (Slattery & Rayner, 2010; Staub, 2020), case alternation (Reingold et al., 2010), or letter rotation (Blythe et al., 2019) reported interaction with word frequency. These interactions are typically stronger in later reading measures such as gaze duration or total reading time.

A plausible theoretical explanation (see Staub, 2020) for this difference is that manipulations such as contrast reduction affect the perceivability of the letter which is located at the feature level of activation-based models (Coltheart et al., 2001; McClelland & Rumelhart, 1981). By contrast, manipulations such as font difficulty or letter rotation affect the spatial configuration of letter features which is located on the letter level (Pelli et al., 2006; Sanocki & Dyson, 2012). Thus, the observed pattern of effects indicates that processing on the feature level is thresholded, while processing on the letter-level directly cascades into linguistic processing on the word-level and increases word frequency effects. One potential mechanism would be that increased processing difficulty on the letter-level necessitates the use of a late checking mechanism in which readers use refixations or regression to resolve uncertainty about letter identity (Staub, 2020). As letter mirroring, similar to letter rotation and font difficulty, clearly changes the spatial configuration of letters features, it is plausible to believe that mirroring and word frequency would produce a similar pattern of interaction.

### Rationale of the present study

**Figure 1.**
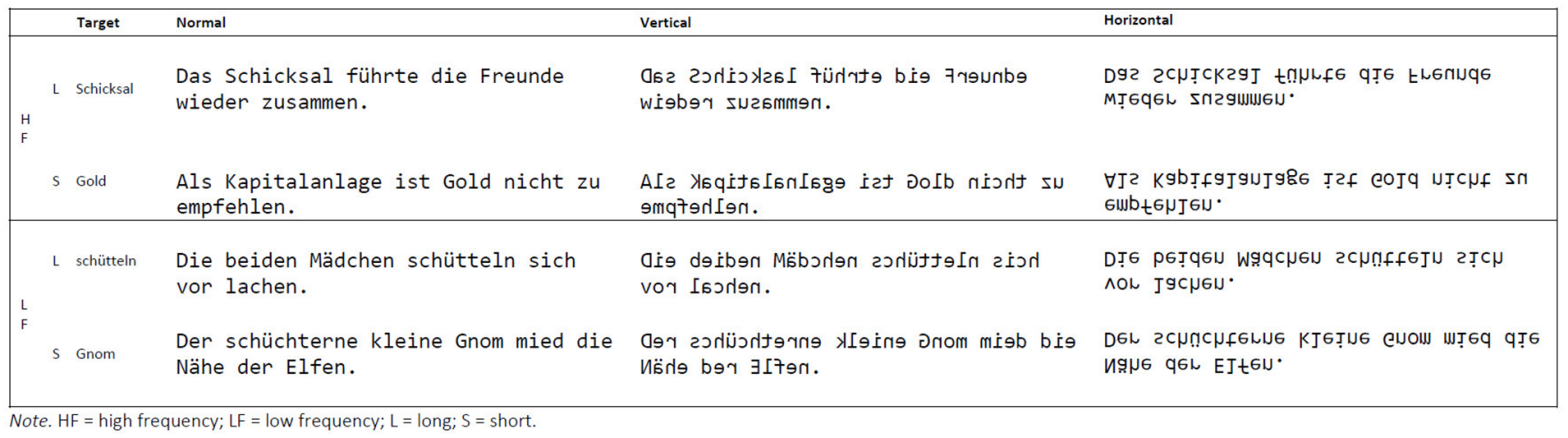
Example stimuli in each of the text presentation conditions.

In the present study, we sought to investigate readers’ eye movements while they were reading mirrored text. To do this, participants read sentences in which the individual letters of a word were either mirrored at their vertical (left-right) or horizontal (up-down) axis. Reading direction was kept intact and words were written from left to right (see Figure 1). Reading materials were the sentences of the Potsdam Sentence Corpus in which target words are orthogonally manipulated for word length and frequency (Kliegl et al., 2004, 2006). Based on previous findings (Chandra et al., 2020; Kolers, 1968; Kowler & Anton, 1987), we expected that both vertical and horizontal mirroring would substantially slow down reading and that horizontal mirroring would be particularly disruptive.

However, we also aimed at investigating the cognitive mechanism that underlies the mirroring effect. In particular, we were interested whether mirror effects are confined to visual processing or whether mirroring also affects lexical stages of the reading process. To address this question we investigated length and frequency effects and their interactions with mirror conditions.

Based on previous single-word recognition studies (Björnström et al., 2014), we expected to see strong length effects in both vertical and horizontal mirrored reading. As horizontal mirroring is generally more disruptive, we also expected that length effects would be stronger here. An interaction between word length and mirror condition would indicate that mirroring effects are visually mediated and mirrored letters are perceptually normalised using a serial, attention-demanding mechanism.

Our predictions for the relationship between mirroring and frequency are less clear. First, it is not obvious whether frequency effects can be observed at all during mirrored reading. Mirroring might be so disruptive that the reading process breaks down completely and participants employ a general problem-solving mechanism that is independent of word frequency. However, given that participants are able to read mirrored text at near-normal reading speed after relatively short training periods, we thought this rather unlikely and expected to see frequency effects in later reading measures. Second, regarding the interaction between mirroring and word frequency, there are two alternative hypotheses. If the two variables have additive effects, this would indicate that mirroring mainly affects early visual processing and leaves lexical processing intact. If, by contrast, the two variables interact with each other, this would indicate that the processing difficulties caused by mirroring on the visual-perceptual level also permeate into later processing stages and interfere with lexical processing.

## Method

### Participants

We recruited 33 participants via the online recruitment system of the University Göttingen. The data of three participants had to be excluded from the analysis due to technical problems or poor data quality. The remaining 30 participants (age: *M* = 22, *SD* = 3 years, 25 women) were native German speakers, had normal or corrected vision, and no record of reading disability. Each individual participant had a performance rate that was > 84%, indicating that participants read sentences accurately. The experiment was conducted in the laboratories of the Department of Educational Psychology and was approved by the ethics committee of the University of Göttingen.

Participants completed a standardised reading fluency subtest (the revised Salzburger Reading and Spelling Test SLRT-II, Moll & Landerl, 2010). Their percentile scores for the word reading subtest, *M* = 59.97, *SD* = 19.78, were slightly higher than the population mean, *t*(29) = 2.76, *p* = .009, whereas their scores on the nonword reading subtest, *M* = 53.97, *SD* = 25.27, were not, *t*(29) = 0.86, *p* = .397.

Results of a post hoc power analysis using the mixed power package (Kumle et al., 2021) revealed that our sample size was adequate to detect with a power of .80 and an α -level of .05 main effects of about 20 ms (first fixation durations), 40 ms (gaze durations), and 51 ms (total reading time); two-way interactions of about 16 ms (first fixation durations), 35 ms (gaze durations), and 37 ms (total reading time) as well as three-way interactions of about 16 ms (first fixation durations), 31 ms (gaze durations) and 35 ms (total reading time).

### Materials

#### The Potsdam Sentence Corpus

We used the Potsdam Sentence Corpus (PSC) as stimulus materials (Kliegl et al., 2004). The PSC consists of 144 German sentences each comprising a target word that was manipulated according to word length and frequency. Frequent words were defined as words having lemma frequencies > 50 fpm (frequency per million) in the CELEX database (Baayen et al., 1995), and infrequent words were defined as words with lemma frequencies below 4 fpm. Word length was is divided into two categories: long words were 6 to 9 letters long and short words were 3 to 5 letters long. Carrier sentences represented a large variety of grammatical structures and the position of the target word ranged from the second to the last word in a sentence (mean target position was 4.9 words).

#### Mirror-fonts

We used the open-source software Font Forge to create customised vertical and horizontal mirror-fonts that were based on the Consolas font. In the vertical mirror condition, letter bitmaps were mirrored at their vertical axis whereas in the horizontal condition letters were mirrored at their horizontal axis. For the horizontal font, bitmaps of individual letters were adjusted to a new baseline. This way we avoided changing the common spatial relationships between adjacent letters (i.e., not aligned: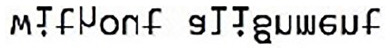). To achieve the correct alignment, we flipped the entire virtual letter-space and created a new imaginary baseline to which the horizontally mirrored letters were aligned (i.e., aligned: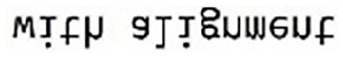).

### Procedure

The study had a 3(Mirror-condition: Normal vs. Vertical vs. Horizontal, within-participant, within-item) × 2(Frequency: High vs. Low, within-participant, between-item) × 2(Length: Long vs. Short, within-participant, between-items) design. Examples for sentences in all conditions are provided in Figure 1. Participants read the sentences silently while their eye movements were being recorded. Each participant read one block in the vertical mirror condition, one block in the horizontal mirror condition, and one block in the normal condition. The order of the three blocks was counterbalanced between participants. In each block, participants read one-third of the sentences of the PSC. Sentences were divided into 3-item lists that were assigned to blocks and participants according to a Latin square design. Each sentence was only read once by each participant. The task of the participants was to read each sentence silently and answer a short comprehension question by pressing the corresponding key on the keyboard. Multiple-choice questions were presented in normal font after 25% of the sentences. For example, the sentence “Even rapeseed can be used to produce fuel” was followed by the question “What can fuel be made from?” with three response alternatives, “flax,” “hemp,” and “rapeseed.”

### Apparatus

An EyeLink 1000 eye tracker (SR Research, Ontario, Canada) was used to record eye movements during reading at a rate of 1000 Hz. Stimuli sentences were presented on a 2100 ASUS LCD monitor, with a refresh rate of 120 Hz. Participants sat at a viewing distance of 65 cm with an assisting head and chin rest to reduce head movements. Sentences were presented in the customised Mirror-Consolas font in black, size 18pt, on a white background using the UMass Eye Track 7.10 m software (Stracuzzi & Kinsey, 2006). Participants used a gamepad to indicate the end of each trial and to provide multiple-choice responses to comprehension questions.

## Results

In a first step, data were cleaned using the popEye package in *R* (Schroeder, 2019). During pre-processing, trials were removed with insufficient calibration quality or too few fixations as well as trials in which a blink occurred directly before or after the target word. In this step, 13.6% of the data were excluded. In addition, we excluded trials with more than 10 runs or 80 fixations. In this step, an additional 3% of the data were excluded.

Data were analysed using generalised linear mixed-effects models with participants and items as crossed random intercepts. Mirror condition was included as a fixed effect in the model and was contrast-coded. For the local measures, word length and frequency were additionally included in the model as contrast-coded fixed effects. The significance of the factors was determined using type III Wald $\chi^{2}$ tests using the ANOVA function of the car package (Fox & Weisberg, 2019). Where necessary post hoc comparisons were conducted using cell means coding and custom-designed contrasts using the *glht* function in the *multcomp* package (Hothorn et al., 2008).

All duration measures were log-transformed prior to the analysis but back-transformed to ease interpretation. In addition, of the log-transformed measures, we excluded all observations deviating more than 2 *SD*s from the person or item mean before the analysis of each measure (excluding 1–1.1% of the data). The results are independent of the specific outlier criterion used for outlier cleaning.

All materials, eye-tracking data, and analysis scripts of this study can be found in the Open Science Framework, at the following URL: https://osf.io/mndb7/?view_only=15f34035449f4c9a90f11c8be0319f7f.

### Global analyses

To examine the effects of mirroring on eye movements on the sentence level we computed average skipping, refixation, and regression probability as well as the number of fixations made on the sentence, mean fixation duration, mean saccade length, first-pass reading duration, rereading time, total reading time, and reading rate. Descriptive statistics and the results from the corresponding linear mixed-effects models are shown in Table 1.

**Table 1.** Model estimates for sentence reading measures in the normal, vertical and horizontal condition and results from linear mixed effects models testing the effect of mirror condition.

	Normal		Vertical		Horizontal		$\chi^{2}$ (1)	p
	M	SE	M	SE	M	SE		
Skipping probability (%)	19.13^a^	0.79	8.70^b^	0.78	4.15^c^	0.78	2,105.58	<0.001***
Refixation probability (%)	20.13^a^	1.36	50.91^b^	1.35	69.98^c^	1.36	6,501.1	<0.001***
Regression probability (%)	9.87^a^	1.09	13.33^b^	1.09	15.47^c^	1.09	137.52	<0.001***
Number of fixations (n)	8.10^a^	0.25	17.12^b^	.54	31.18^c^	0.98	11,713.2	<0.001***
Mean Fixation Duration (ms)	215^a^	4	294^b^	6	355^c^	7	8,796.6	<0.001***
Saccade length (n letters)	6.85^a^	0.21	4.26^b^	0.13	2.95^c^	0.09	12,752.0	<0.001***
Firstpass duration (ms)	1575^a^	69	3941^b^	172	3941^c^	346	16,461	<0.001***
Re-reading time (ms)	8^a^	2	82^b^	17	266^c^	55	756.14	<0.001***
Total sentence reading time (ms)	1746^a^	75	5040^b^	2016	10827^c^	465	17,071	<0.001***
Reading rate (words/min)	267.18^a^	11.11	92.71^b^	3.84	43.47^c^	1.81	17,109	<0.001***

*Note*. Different letters indicate significant contrast.

* p < .05, **p < .01, ***p < .001.

Results showed a main effect of mirror condition on all global eye-movements measures. Post hoc contrasts between the normal and the vertical condition revealed a significant increase in re-fixation probability (*SE* = 0.006, *z* = 51.11, *p* < .001), regression probability (*SE* = 0.005, *z* = 7.451, *p* < .001), number of fixations (*SE* = 0.012, *z* = 61.76, *p* < .001), mean fixation duration (*SE* = 0.005, *z* = 60.51, *p* < .001), firstpass duration (*SE* = 0.012, *z* = 76.14, *p* < .001), re-reading time (*SE* = 0.125, *z* = 18.43, *p* < .001) and total sentence reading time (*SE* = 0.013, *z* = 78.73, *p* < .001). By contrast, mean saccade length (*SE* = 0.007, *z* = –66.33, *p* < .001), skipping probability (*SE* = 0.003, *z* = –32.29, *p* < .001) and reading rate (*SE* = 0.013, *z* = –79.18, *p* < .001) were lower in the vertical than in the normal condition.

Furthermore, reading times differed significantly between the horizontal and the vertical condition for re-fixation probability (*SE* = 0.006, *z* = 31.37, *p* < .001), regression probability (*SE* = 0.005, *z* = 4.469, *p* < .001), number of fixations (*SE* = 0.012, *z* = 49.04, *p* < .001), mean fixation duration (*SE* = 0.005, *z* = 35.74, *p* < .001), first-pass duration (*SE* = 0.012, *z* = 56.46, *p* < .001), re-reading time (*SE* = 0.127, *z* = 9.283, *p* < .001), and total sentence reading time (*SE* = 0.014, *z* = 55.86, *p* < .001). Again, this pattern was reversed for saccade length (*SE* = 0.007, *z* = –50.45, *p* < .001), reading rate (*SE* = 0.014, *z* = –55.63, *p* < .001), and skipping probability (*SE* = 0.003, *z* = –14.781, *p* < .001).

In sum, in line with previous findings, reading in both mirror conditions was more difficult than in the normal condition. Reading the sentences in the PSC in the normal condition took about 1.7 s, in the vertical mirror condition about 5 s, and in the horizontal condition about 10 s. Also in line with previous studies, reading in the horizontal condition took approximately twice as long than in the vertical mirror condition. Similar effect sizes have been reported by Kolers (1968). However, effects are substantially smaller than the effects reported by Kowler and Anton (1987). Beyond replicating the overall effects of mirroring on total reading time, our findings show that mirroring has both immediate (skipping probability, mean fixation duration, first-pass reading time) and delayed effects (regression rate, re-reading time, and regression probability). We will come back to this finding in the discussion section.

### Local analyses

**Figure 2.**
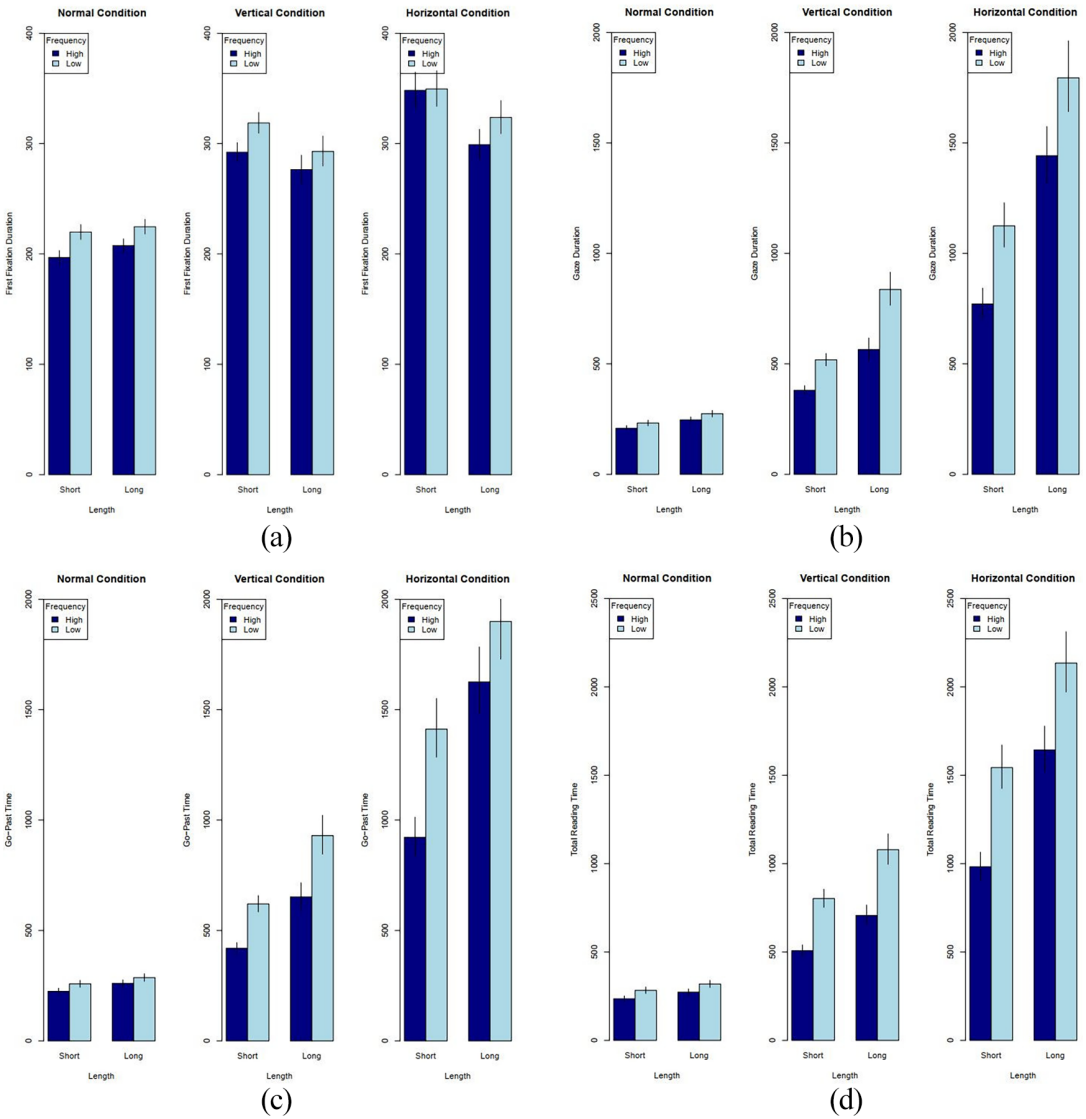
Plots a-d: First Fixation Duration, Gaze Duration, Go-past Time and Total Reading Time. (a) First Fixation Duration (b) Gaze Duration (c) Go-past Time (d) Total Reading Time.

To examine the effects of horizontal and vertical mirroring on the target words which were manipulated according to word length and frequency, we computed five dependent measures: first fixation duration, gaze duration, go-past time, and total reading time. Descriptive statistics for all measures are provided in Table 2. The results of the corresponding linear mixed-effects models are reported in Table 3 and depicted in Figure 2. Prior to the analysis, we excluded all target words with more than 14 fixations or that were reread for more than 4 times (excluding ca. 1.7% of the data). In addition, we removed outlying observations deviating more 2 *SD*s from the person or item mean before the analysis of each measure (excluding 1–6% of the data).

**Table 2.** Model estimates for first fixation duration, gaze duration, go-past time, and total reading time (ms) to target Words (*SE*s in parentheses).

	Frequency	Normal		Vertical		Horizontal	
		Long	Short	Long	Short	Long	Short
First fixation duration	HF	222 (8)	196 (7)	306 (10)	284 (9)	346 (18)	350 (18)
	LF	226 (8)	210 (7)	289 (15)	275 (14)	314 (16)	299 (15)
Gaze Duration	HF	230 (9)	204 (8)	516 (20)	380 (15)	1,124 (121)	767 (83)
	LF	264 (10)	237 (9)	844 (91)	567 (61)	1,795 (189)	1,370 (147)
Go-past Time	HF	247 (12)	214 (11)	629 (31)	408 (20)	1,354 (131)	916 (89)
	LF	270 (13)	246 (12)	927 (89)	641 (62)	1,920 (185)	1,566 (149)
Total Reading Time	HF	265 (12)	225 (11)	798 (37)	496 (23)	1,568 (126)	954 (77)
	LF	302 (14)	257 (12)	1,078 (87)	704 (57)	2,130 (172)	1,607 (128)

HF: high frequency; LW: low frequency; *SE*: standard error.

**Table 3.** Results from linear mixed-effects models ( $\chi^{2}$ Wald tests) for first fixation duration, gaze duration, go-past time, and total reading time.

	FFD	GD	GPT	TRT
Intercept	98,225.94***	32,198.03***	26,492***	27,755.61***
Mirror-condition	447.72***	282.48***	394.09***	549.55***
Length	8.03***	112.77***	49.01***	26.92***
Length × Mirror-condition	18.44***	39.20***	31.86***	22.37***
Frequency	16.28***	31.36***	31.13***	33.43***
Frequency × Mirror-condition	1.92	17.62***	17.14***	16.67***
Length × Frequency	0.02	0.08	1.56	0.58
Length × Frequency × Mirror-condition	2.40	2.85	3.95	2.16

FFD: first fixation duration; GD: gaze duration; GPT: go-past time; TRT: total reading time.

* *p* < .05, **p < .01, ***p < .001.

#### First fixation duration

There was a main effect of mirror condition: First fixation durations for words in the vertical condition, *M* = 288 ms, *SE* = 11 ms, were ∆b = .303 (75 ms) longer *z* = 10.570, *p* < .001, than in the normal condition, *M* = 213 ms, *SE* = 6 ms. Similarly, first fixation durations in the horizontal condition, *M* = 326 ms, *SE* = 14 m, were ∆b = .427 (113 ms) longer than in the normal condition, *z* = 10.880, *p* = < .001. The contrast between the horizontal and the vertical condition was also significant, *z* = 4.153, *p* = < .001.

Furthermore, there was main effect of length. First fixation durations for short words, *M* = 278 ms, *SE* = 11 ms, were ∆b = –0.043 (12 ms) longer, *z* = –2.707, *p* = .006, than for long words, *M* = 266 ms, *SE* = 9 ms. In addition, the interaction between word length and mirror condition was significant, indicating that length effects were more pronounced in the two mirror conditions than in the normal condition. In particular, the length effect in the normal condition, ∆b = .042 (9 ms), *z* = 2.195, *p* = .028, was significantly smaller, *z* = –3.001, *p* = .002, than the length effect in the vertical condition, ∆b = –0.043 (13 ms), *z* = –1.981, *p* = .048, and also significantly smaller, *z* = –4.364, *p* < .001, than the length effect in the horizontal condition ∆b = –0.128 (42 ms), *z* = –3.819, *p* < .001. The length effects in the vertical and horizontal conditions differed significantly from each other, *z* = 2.373, *p* = .018.

Notice that length effects in the two mirror conditions were inverted, i.e., short words were processed *slower* than long words, while shorter words in the normal condition were processed faster than long words. The inverted length effects can be explained by a trade-off between first fixation duration and refixation probability. That is participants were much more likely to refixate words in the two mirror conditions (see Table 1), but, as a consequence, each of the individual fixations was shorter.

There was also a main effect of frequency: First fixation durations for frequent words, *M* = 264 ms, *SE* = 9 ms, were ∆b = –0.060 (16 ms) shorter than for infrequent words, *M* = 280 ms, *SE* = 9 ms. The interaction term for frequency and mirror condition was not significant.

#### Gaze duration

There was a main effect of mirror condition: Gaze durations for words in the normal condition, *M* = 233 ms, *SE* = 7 ms, were ∆b = .866 (320 ms) shorter than gaze durations for words in the vertical condition, *M* = 553 ms, *SE* = 39 ms, *z* = 15.99, *p* < .001, which were in turn ∆b = .773 (646 ms) shorter than in the horizontal condition, *M* = 1199 ms, *SE* = 110, *z* = 12.27, *p*  < .001.

Furthermore, there was a main effect of length. Gaze durations for short words *M* = 447 ms, *SE* = 32 ms, were ∆b = .367 (197 ms) shorter than for long words, *M* = 644 ms, *SE* = 40 ms. In addition, the interaction between word length and mirror condition was significant, indicating that length effects were more pronounced in the two mirror conditions than in the normal condition: In the normal condition, the length effect ∆b = .143 (40 ms), *z* = 4.786, *p* < .001, was significantly shorter, *z* = 5.003, *p*  < .001, than the length effect in the vertical condition, ∆b = .447 (249 ms), *z* = 6.732, *p* < .001 and also significantly shorter, *z* = 5.646, *p* < .001, than the length effect in the horizontal condition, ∆b = .511 (620 ms), *z* = 7.230, *p* < .001. The length effects in the horizontal and the vertical condition did not differ significantly from each other, *z* = –0.826, *p* = .409.

There was a main effect of frequency. Gaze durations for frequent words *M* = 471 ms, *SE* = 29 ms, were ∆b = –.260 (138 ms) shorter than gaze durations for infrequent words, *M* = 611 ms, *SE* = 38 ms, *z* = –6.002, *p* < .001. The interaction of frequency and mirror condition was also significant: The simple frequency effect in the normal condition, ∆b = –0.114 (26 ms), *z* = –3.836, *p* < .001, was substantially smaller, *z* = 5.607, *p* < .001, than the simple frequency effect in the vertical condition, ∆b = –0.352 (195 ms), *z* = –5.301, *p* < .001, and substantially smaller, *z* = 4.924, *p* < .001, than the frequency effect in the horizontal condition, ∆b = –0.313 (377 ms), *z* = –4.427, *p* < .001. In addition, the frequency effect in the vertical mirror condition was significantly smaller than in the horizontal mirror condition, ∆b = .664 (182 ms), *z* = 5.899, *p*  < .001, indicating that frequency effects were more pronounced in the horizontal compared with the vertical mirror condition.

#### Go-past time

There was a main effect of mirror condition: Go-past time for words in the normal condition, *M* = 243 ms, *SE* = 10 ms, was ∆b = .943 (382 ms) shorter than go-past time for words in the vertical condition, *M* = 625 ms, *SE* = 51 ms, *z* = 17.55, *p* > .001, which in turn was ∆b = .799 (765 ms) shorter than in the horizontal condition, *M* = 1390 ms, *SE* = 97, *z* = 16.48, *p*  < .001.

Furthermore, there was a main effect of length. Go-past time for short words, *M* = 506 ms, *SE* = 39 ms, was ∆b = .326 (195 ms) shorter than for long words, *M* = 701 ms, *SE* = 46 ms. In addition, the interaction between word length and mirror condition was significant, indicating that length effects were more pronounced in the two mirror conditions than in the normal condition: In the normal condition, the length effect ∆b = .115 (28 ms), *z* = 3.666, *p* < .001, was “significantly shorter”, *z* = 4.633, *p*  < .001, than the length effect in the vertical mirror condition, ∆b = .421 (265 ms), *z* = 5.691, *p* < .001, and also significantly shorter, *z* = 4.586, *p* < .001, than the length effect in the horizontal mirror condition, ∆b = .443 (621 ms), *z* = 5.423, *p* < .001. The length effects in the horizontal and the vertical condition did not differ significantly from each other, *z* = –0.323, *p* = .746.

There was a main effect of frequency. Go-past time for frequent words *M* = 520 ms, *SE* = 34 ms, were ∆b = –0.272 (162 ms) shorter than go-past time for infrequent words, *M* = 682 ms, *SE* = 45 ms, *z* = –5.17, *p* < .001. The interaction of frequency and mirror condition was also significant: The simple frequency effect in the normal condition, ∆b = –0.119 (29 ms), *z* = –3.812, *p* < .001, was substantially smaller, *z* = 5.629, *p* < .001, than the simple frequency effect in the vertical condition, ∆b = –0.401 (253 ms), *z* = –5.423, *p* < .001, and substantially smaller, *z* = 4.127, *p* < .001, than the simple frequency effect in the horizontal condition, ∆b = –0.297 (414 ms), *z* = –3.641, *p* < .001; In addition, the frequency effect in the vertical condition was significantly smaller than in the horizontal mirror condition, ∆b = .699 (161 ms), *z* = 5.014, *p*  < .001, indicating that frequency effects were more pronounced in the horizontal compared with the vertical mirror condition.

#### Total reading time

There was a main effect of mirror condition: Total reading time for words in the normal condition, *M* = 261 ms, *SE* = 9 ms, was ∆b = 1.043 (479 ms) shorter than total reading time for words in the vertical condition, *M* = 740 ms, *SE* = 40 ms, *z* = 24.73, *p* < .001, which in turn was ∆b = .709 (764 ms) shorter than total reading time in the horizontal condition, *M* = 1504 ms, *SE* = 83, *z* = 16.1, *p* < .001.

Furthermore, there was a main effect of length. Total reading time for short words, *M* = 573 ms, *SE* = 32 ms, was ∆b = .290 (193 ms) shorter than total reading time for long words, *M* = 766 ms, *SE* = 37 ms. In addition, the interaction between word length and mirror condition was significant, indicating that length effects were more pronounced in the two mirror conditions than in the normal condition: In the normal condition, the length effect ∆b = .131 (34 ms), *z* = 3.599, *p* < .001, was significantly shorter, *z* = 2.807, *p* = .005, than the length effect in the vertical mirror condition ∆b = .325 (242 ms), *z* = 4.112, *p* < .001, and also significantly shorter, *z* = 4.626, *p* < .001, than the length effect in the horizontal mirror condition ∆b = .414 (627 ms), *z* = 5.817, *p* < .001. The length effect in the vertical mirror condition did not differ significantly from the length effect in the horizontal mirror condition, ∆b = –0.089 (385 ms), *z* = –1.369, *p* = .171.

Furthermore, there was a main effect of frequency. Total reading time for frequent words, *M* = 560 ms, *SE* = 27 ms, was ∆b = –0.335 (46 ms) shorter than for infrequent words, *M* = 783 ms, *SE* = 38 ms, *z* = –6.323, *p* < .001. The interaction of frequency and mirror condition was also significant. The simple frequency effect in the normal condition, ∆b = –0.163 (43 ms),*z* = –4.488, *p* < .001, was substantially smaller, *z* = 6.034, *p* < .001, than the simple frequency effect in the vertical mirror condition, ∆b = –0.451 (337 ms), *z* = –5.704, *p* < .001, and the simple frequency effect in the horizontal mirror condition, ∆b = –0.389 (589 ms), *z* = –5.467, *p*  < .001; In addition, the frequency effect in the vertical condition was significantly smaller than in the horizontal mirror condition, ∆b = .840 (252 ms), *z* = 6.190, *p*  < .001, indicating that frequency effects were more pronounced in the horizontal compared with the vertical mirror condition.

## Discussion

This study investigated the cognitive mechanism that underlies the reading of text with mirrored letters. To this end, we recorded the eye movements of skilled adult readers as they read the Potsdam Sentence Corpus (PSC) (Kliegl et al., 2004). Individual letters were mirrored horizontally (around their up-down axis) or vertically (around their left-right axis). Each sentence of the PSC comprised a target word that is manipulated for length and frequency. We were particularly interested in length and frequency effects and their interaction with mirror condition. Our main findings are that reading horizontally mirrored text disrupted the reading process more than reading vertically mirrored text. In addition, mirror condition interacted with word length and word frequency. Below we elaborate on our key findings and explain their theoretical implications.

First, we found that reading mirrored text substantially slows down the reading process. Based on past research with mirrored text (Chandra et al., 2020; Kolers, 1968; Kowler & Anton, 1987), we expected that sentences with vertically mirrored letters would produce less disruption in both global and local eye movement measures relative to sentences with horizontally mirrored letters. This expectation was confirmed for all early and late measures examined in this study. Based on the previous mirror reading studies with vertically and horizontally mirrored text, we expected vertical mirroring to be less disruptive than horizontal mirroring (Kolers, 1968).

Furthermore, past studies from single-word recognition have shown that reading words with mirrored letters substantially increases word length effects (Björnström et al., 2014). Thus, we expected (1) that word length effects in both mirror conditions would be more pronounced than in the normal condition and (2) that the size of the word length effect would be larger in the horizontal than in the vertical mirror condition. The first hypothesis was confirmed. We found that length effects were significantly more pronounced in each mirror condition compared with the normal condition for all late eye-movement measures in the local analysis of the target words: gaze duration, go-past time, and total reading time. By contrast, the second hypothesis was not confirmed. Length effects were equally pronounced in both mirror conditions. Our results are thus in line with the assumption that readers apply attention-demanding serial letter-by-letter decoding strategy in mirror reading (Cohen et al., 2008). This effect, however, is independent of the direction of mirroring. Overall, our results thus show that mirror reading is fundamentally similar to other kinds of visual manipulations which slow down reading by enforcing serial letter-by-letter encoding. It would be interesting to see whether similar interactions with word length would occur for other visual manipulations typically used in eye-tracking studies (stimulus degradation, font difficulty, and letter rotation; Blythe et al., 2019; Staub, 2020).

Turning to the effects of word frequency, a first important finding is that we observed substantial frequency effects for both early (first fixation duration) and later (gaze duration, go-past time, and total reading time) eye-movement measures. This indicates that language-related, lexical processes are intact during reading mirrored text and participants did not identify words using a general problem-solving strategy that is unrelated to reading. Thus, participants read the mirrored text more slowly, but they were still reading.

In addition, we found that mirroring and word frequency interacted in gaze duration, gopast time, and total reading time, i.e., the size of the frequency effect was larger in both mirror conditions than in the normal condition. This pattern is similar to the findings of Staub (2020) who also reported interacting effects of word frequency and font difficulty manipulation for later reading measures. Similarly, Blythe et al. (2019) reported an interaction between letter rotation and word frequency for gaze duration and go-past time. Moreover, our findings indicate that frequency effects were larger in the horizontal than in the vertical mirror condition. This indicates that uncertainty about letter identity was higher in the horizontal condition and, as a consequence, it is more important to verify the correct lexical interpretation later in the reading process (Staub, 2020).

Turning to the theoretical implications of our findings, our results are informative about the relationship between the letter-level and the word-level of processing. Mirroring effects are similar to other visual manipulations such as font difficulty or letter rotation which are located on the letter level and which have previously been shown to produce interactive effects with word frequency. The interaction of mirroring and frequency indicates that difficulties in letter processing permeate to the word level where they produce the observed larger frequency effects. This supports the notion that processing is cascaded between the letter and the word level. The finding that these interactions were only observed for later reading measures implicates some corrective process on the word level, such as an additional checking mechanism during lexical verification.

In the present study, we showed *when* mirroring affects the reading process. Further research will be required to address the question of *why* these interference effects occur. Potential mechanisms are:

1. Mirrored letters consist of visual features that are less frequent or familiar to the reader.
2. Letter features are combined differently in mirrored letters and, as a consequence, new feature-letter mappings have to be established.
3. Mirroring distorts supra-letter information such as word shape or frequently co-occurring feature combinations.
4. Mirror reading can create interference effects if the mirror image of a letter is similar to a different letter in normal orientation (e.g., “b” and “d”).

These explanations are not mutually exclusive and mirror effects are likely to be a result of several mechanisms at the same time. In addition, the different mechanisms might contribute differentially to vertical and horizontal mirroring effects. For example, vertical mirroring is more likely to preserve word shape information than horizontal mirroring. To systematically investigate the various mechanisms, future research should manipulate these different features separately. Because in our study length and frequency were between item manipulations and our sample size might have been not sufficient to detect the triple interactions between length, frequency, and mirror condition, it would be helpful to replicate our findings using a within items design in a larger eye movement experiment. In addition, future studies should investigate how additionally changing the order of the letters in a word or mirroring the word as a whole (“word” vs. “drow”) affects the reading process (e.g. Chandra et al., 2020; Kolers, 1968).

## Conclusion

This study adds to the body of research exploring how letter mirroring affects the reading processes. We extend previous work on the mirroring effect which has primarily focused on reading on the text- or word-level. We recorded participants’ eye movements while they read single sentences in which target words were manipulated according to their length and frequency. Furthermore, we extended previous eye-movement research on the mirroring effect in that we additionally included horizontally mirrored letters in our experimental design.

Our results show that on the sentence level, reading a horizontally mirrored script is substantially more disruptive than reading a vertically mirrored script. In addition, both mirror conditions did substantially increase word length effects compared to the normal condition, indicating that participants processed words more serially during mirror reading. However, frequency effects were observed in all reading measures showing that lexical processing was still intact. In addition, mirror condition and word frequency interacted which indicates that mirroring affected language-related processes.
